# Is the Dark Triad Better Studied Using a Variable- or a Person-Centered Approach? An Exploratory Investigation

**DOI:** 10.1371/journal.pone.0161628

**Published:** 2016-08-31

**Authors:** Chester Chun Seng Kam, Mingming Zhou

**Affiliations:** 1 University of Macau, Taipa, Macau, China; 2 The University of Western Ontario, London, Ontario, Canada; TNO, NETHERLANDS

## Abstract

Despite Allport’s early call to study personality as a coordinated system of traits within individual rather than separate traits, researchers often assume personality variables are largely distinct, independent characteristics. In the current research, we examined the usual assumption that Dark Triad traits (narcissism, psychopathy, and Machiavellianism) are best studied using a variable-centered (dimensional), rather than a person-centered (taxonic), approach. Results showed that a variable-centered approach is appropriate in understanding the Dark Triad, and yet individuals scoring high on one Dark Triad dimension also tend to score high on other dimensions. Based on these results, we concluded that it is appropriate to study individual differences in the Dark Triad (inferences based on persons) by capturing the common variance among the three traits using a variable-centered approach, rather than treating these traits as independent or uncoordinated characteristics.

## Introduction

Drawing on apparently distinct research traditions, Paulhus and Williams [1] grouped three personality concepts that capture individual differences in malevolent qualities. These concepts were collectively named “the Dark Triad.” Upon publication of Paulhus and Williams [1] the concepts have gained widespread interests, as seen in the large number of empirical investigations of their utility in predicting human behavior [2–6]. Later, as an important piece of research in this area, Jonason, Li, Webster, and Schmitt [7] extracted the common variance among the three concepts to capture the Dark Triad, potentially implying that measures for the three concepts represent a composite. Some subsequent work have followed the same methodological practice [8–10].

The concepts included in the Dark Triad are the subclinical traits of narcissism, psychopathy, and Machiavellianism. Narcissistic individuals are characterized by their highly positively inflated but unrealistic self-views [11]. This excessive self-aggrandizement is usually maintained by intrapsychic (e.g., fantasizing about power; [12]) and interpersonal strategies (e.g., using their relationships in the service of the self; [11]). Psychopathy is described as a personality and developmental disorder that features reduced guilt, impaired empathy, and salient antisocial behaviors [13, 14]. People high in psychopathy tend to be impulsive, non-empathetic, egocentric, with minimal emotional responsiveness to threatening stimuli [1, 15, 16]. Machiavellianism denotes the tendency to exploit others to one’s own advantage in a manipulative, cynical, and insincere interpersonal manner [17–19]. Individuals with high levels of Machiavellianism tend to employ exploitative tactics [20], by adopting strategies that maximize self-interest [21].

As pointed out by past researchers such as Foster and Campbell [22], scholars in clinical and personality fields adopted different approaches to examine pathological personality traits. Personality psychologists took a variable-centered (or dimensional) approach in the study of personality, assuming the variables as continuous [22], such as isolating the influence of one continuous variable from another on predicting the outcome variables or the interaction of two or three variables together on an outcome. With this approach, there is no uniform cutoff score on a continuous variable that differentiates normal from pathological individuals, and individuals do not differ in type, but only in degree.

In contrast, clinical psychologists typically took a person-centered (or taxonic) approach to personality disorders [23], with the main goal of developing diagnostic criteria in order to identify individuals at risk of psychological conditions, classify individuals into groups, and prescribe appropriate treatments based on group memberships [24–26]. In trait research related to the Dark Triad, narcissism and psychopathy have a strong tradition as categorical variables, suggesting that within each of these conditions individuals are classified as members of qualitatively distinct subgroups (e.g., [27,28] for narcissism; [25, 29–32] for psychopathy).

When personality psychologists extended the examination of these pathological traits from special population (e.g., criminals) to normal population (e.g., students), they did not necessarily adopt the idea that these conditions, in themselves or in combination of each other, are taxonic in nature, but assumed them as dimensional constructs [33]. Typically, personality researchers adopt the Dark Triad as three distinct variables. They then examine the relationship between each measure and its potential antecedents, correlates, and consequences [34–36]. This practice was also observed when scholars examined the Dark Triad with a neurological perspective. Recent findings through neurobiology theories (e.g., Reinforcement Sensitivity Theory, Gray [37] revealed that all three Dark Triad traits were respectively positively correlated with negative affectivity, reward sensitivity, and dysfunctional impulsivity [38]. Although less common, some researchers treat the three Dark Triad constructs as one [7], averaging to create a composite Dark Triad score [39]. This approach is supported by empirical studies demonstrating that the Dark Triad constructs load on a single latent factor [7, 40–42].

Without criticizing any of the previous studies, we are aware of no empirical or theoretical basis on which the structure of the Dark Triad should be dimensional or taxonic in nature and whether Dark Triad should be studied using variable-centered or person-centered approach. Statistically, a critical assumption of the variable-centered approach is the underlying homogeneity of the population regarding the construct's trait structure [43]. If a causal relationship exists between the predictors (such as the Dark Triad) and the outcome (such as psychological health), the process is assumed to be universal among members of the same population [44, 45]. In other words, members of the same population are only *quantitatively* different from each other; the process of how they relate to the outcome is *qualitatively* similar [46].

If the assumption of population homogeneity is violated, a variable-centered approach will not be able to provide an accurate picture of the analytic findings [47]. Indeed, many psychological conditions, such as anxiety and borderline personality traits, have been empirically shown that they were better operationalized using person-centered approaches (i.e., individuals can be classified into different subtypes of the same disorder [48]), although empirical research still severely lags behind in discovering potential subtypes within other clinical or personality traits.

On the conceptual level, personality characteristics may not always be best studied using a variable-centered approach. As pointed out by Allport [49], different psychological processes, behaviors, and traits function as a coordinated system, rather than isolated from each other. If we wish to examine personality constructs, a person-centered perspective may be desirable, with the unit of analysis being the person, not a trait (see Asendorpf [33], for an insightful discussion). Unfortunately, researchers often assume personality variables are distinct entities from each other [33], with the occasional exception that two or more variables are studied as interacting entities (i.e., moderation) to predict an outcome. Following Allport’s [49,50] advice, we feel the necessity to examine how to best approach Dark Triad variables rather than assuming the Dark Triad traits are three distinct yet related constructs.

### Present research

The purpose of the current research is to re-visit the research question of whether any qualitatively distinct groups exist in the Dark Triad. If the answer is affirmative, the Dark Triad should be operationalized using person-centered (taxonic) approach rather than variable-centered (dimensional) approach. Previous findings based on variable-centered approaches would be questionable to the extent that the operationalization of the construct does not accurately reflect its latent structure. Admittedly, there is a lack of theoretical work investigating the potential structure of the Dark Triad (whether it should be taxonic or dimensional), despite the fact that knowing its structure is important for its future theory development. Although some researchers prefer an empirical investigation driven by substantial theorization, in situations when this is not feasible, having empirical findings to guide future theory on the Dark Triad is often beneficial. This approach of scientific investigations can be found on the structure of personality traits [51], which was discovered using primarily factor analytic techniques rather than substantial theory development. Therefore, we make no specific hypothesis on the structure of the Dark Triad because few previous studies [52] were conducted using person-centered approaches. With a rather small sample size and subjective criteria (by examining how interpretable a cluster analytic solution is), the data of Chabrol et al. [52] seem to support a taxonic, person-centered approach in the study of the Dark Triad and a related trait, although their results are perhaps suggestive in nature. Therefore, because of insufficient research in this area, we have employed a larger sample size and will allow statistical analyses to provide the answer regarding the best approach to study Dark Triad traits.

Confirmatory factor analysis (CFA) is a variable-centered approach by which researchers can specify the hypothetical factor structure a priori and determine the best-fitting factor structure [53]. CFA extracts the common, shared variance among a set of observed indicators, such as participants’ scores on Dark Triad constructs. Variable-centered approaches such as CFA are best employed when individuals differ only quantitatively (i.e., individuals without distinct Dark Triad profiles). Latent profile analysis (LPA), in contrast, is a person-centered approach that shifts the focus of investigation from variables to individuals. LPA aims to identify groups of individuals with unique profile characteristics (e.g., one group may be high on all three constructs, whereas another may be high on one or two). Compared to traditional methods of person-centered research (e.g., cluster analysis), LPA has several advantages: (a) LPA uses maximum likelihood estimation to obtain estimated probabilities of class membership; (b) LPA employs latent variable modeling to reduce measurement error; and (c) LPA calculates fit indices to facilitate model selection [54].

A person-centered approach best represents the data if individuals differ on profile characteristics. However, person-centered approaches alone assume homogeneity within each group (i.e., members assigned to the same group do not differ), which may be unrealistic. Therefore, we also employed factor mixture modeling (FMM), which relaxes the assumption of within-group homogeneity in LPA. Individuals assigned to a group are allowed to differ quantitatively. In this sense, FMM analysis is a “best-of-both-worlds” approach, combining the benefits of variable- and person-centered approaches.

The ultimate solution will be determined jointly by fit indices and profile shape offered by CFA, LPA, and FMM. Fit indices provide an objective measure of the soundness of a solution, but relying solely on them invites the problem of overfitting the data. Therefore, we take the following approach: If the LPA or FMM solutions reveal profiles with qualitatively distinct shapes, they would support the existence of categorically or qualitatively heterogeneous groups of individuals in the data (person-centered approach). If the solutions show profiles parallel to each other, it would suggest only quantitative differences (variable-centered approach). Finally, to examine if personal-centered approach may show a substantial advantage above and beyond variable-centered approach in the study of the Dark Triad, we conducted a nomological network investigation by correlating the Dark Triad with external constructs: Big Five and social dominance orientation (SDO). Big five and SDO were chosen because they are common correlates of Dark Triad traits [10]. If person-centered approach shows additional value beyond the variable-centered approach, the former may be able to reveal non-linear or non-stepwise relations with external correlates (e.g., all profiles share low SDO or agreeableness values except the most severe class of Dark Triad). To ensure the quality of our respondents’ answers, we included additional items requesting participants to select a particular response in our survey. Those who failed to follow our instructions were regarded as potential careless respondents and excluded from our analyses.

## Method

### Participants

Data collection was approved by the Institutional Review Board (IRG) of the University of Western Ontario. We employed a large sample size, as required for LPA analysis. Participants were 1,406 undergraduates at a Canadian university in southwestern Ontario who completed a survey in exchange for partial course credit. Of the entire sample, 1,102 participants (78.38%) passed two check questions that requested them to select a particular response in the survey (‘*Strongly Agree*’ for one question and ‘*Disagree*’ for another question). This type of question has been found to be helpful in screening out careless respondents [55, 56]. We included the 1,102 careful respondents so that our solution would not be distorted by inattentive responding. Finally, this dataset has been used in other research unrelated to the current study.

### Measures

Participants completed survey questions about the Dark Triad, together with two other measures: Big Five personality and Social Dominance Orientation. There has long been argument that the Dark Triad consisted of a group of personality traits [57, 58], and it had the same common core as social dominance orientation [9]. Nonetheless, these arguments were examined only from a variable-centered perspective. We decided to include these additional measures in order to find out if the person-centered approach will show additional value.

#### Dark Triad

The Dark Triad was assessed by a short-form measure [59] of Machiavellianism (α = .75), subclinical psychopathy (α = .77), and subclinical narcissism (α = .67; Cronbach’s alphas in the current study are shown in parentheses). Each subscale was measured in a 5-point Likert scale (1 = *Strongly Disagree*; 5 = *Strongly Agree*). The entire measure consisted of 28 items: Machiavellianism was measured by 10 items, and narcissism and psychopathy were each measured by 9 items. Sample items were “It’s not wise to tell your secrets” (Machiavellianism); “I like to get revenge on authorities” (psychopathy); and “I know that I am special because everyone keeps telling me so” (narcissism). Paulhus and Jones [59] also found their scales to have acceptable level of reliability in their study (αs > .73). In the current study, the inter-correlations among the three traits were not strong (all *p*s < .001): Machiavellianism and psychopathy: .50; Machiavellianism and narcissism: .20; narcissism and psychopathy: .23. The current version of the Dark Triad has been used in recent studies (e.g., [60–64]).

#### Big Five personality measures

Participants completed the Big Five personality measures (NEO domain) from the International Personality Item Pool [65]. The scale measures openness (α = .73), conscientiousness (α = .80), extraversion (α = .88), agreeableness (α = .76), and neuroticism (α = .86). Each personality construct was measured by 10 items in a 5-point Likert scale (1 = *Strongly Disagree*; 5 = *Strongly Agree*).

#### Social dominance orientation (SDO)

Participants completed 16 items measuring their preference toward social group inequality [66], such as the legitimacy of superior groups dominating other groups. The scale (α = .90) was measured in a 7-point Likert scale (1 = *Strongly Disagree*; 7 = *Strongly Agree*).

### Analysis strategies

The current study compares the results of person-centered (LPA) and variable-centered (CFA) approaches. The analyses were conducted in Mplus 7.2, with robust maximum likelihood estimator. An advantage of using a robust maximum likelihood estimator was the relaxation of strict multivariate normality as a requirement for data analysis. In addition, the Mplus program provided a list of comprehensive indices for model selection.

Following common practice, we calculated the scores of psychopathy, Machiavellianism, and narcissism by averaging scores of their corresponding items, and using these three scores as construct-level indicators in LPA analysis. LPA is very demanding in its sample size requirement. Using these construct-level indicators (as opposed to individual item scores) can decrease the number of parameters being estimated and thus alleviate the burden in achieving a stable solution.

In CFA, we constructed a model in which the scores of psychopathy, Machiavellianism, and narcissism all load onto one latent (continuous) variable. In LPA, we started with a two-profile solution, then gradually increased the number of profiles in the latent variable (i.e., the Dark Triad). A two-profile solution was conducted with 1,000 random starts with 60 iterations, in which the 250 best solutions were retained for final stage optimization. As the model becomes more complex (i.e., the number of profiles increases), the random starts increase to 2,000, with the 500 best solutions further analyzed for optimization. As LPA is known to have the problem of local maxima, all final solutions were checked to ensure successful replication of the best solution with different seeds (i.e., global maximum). Finally, the default of Mplus programme restricts the variance of indicators to be identical across profiles (e.g., in a 3-profile solution, the variance of psychopathy is constrained to be the same across all three profiles), but this assumption has been shown to be too restrictive [67], and there is usually no theoretical reason behind this assumption. Following the advice of Morin, Maïano, Marsh, Janosz, and Nagengast [68, 69], we lifted this restriction and freely estimated the variances in each profile.

In model selection, both CFA and LPA models provided a number of loglikelihood-based fit indices for this purpose, including the Akaike’s Information Criterion (AIC), the Bayesian Information Criterion (BIC), and the sample-size adjusted BIC (SABIC). A lower value of these indices indicates a better solution. Although there is no infallible rule in model selection, recent simulations show that BIC performed the best at identifying the correct model, compared to many other fit indices [70, 71].

Among latent profile models, there is additional fit information to facilitate model selection. Entropy informs the researchers about classification certainty among the participants. Ranging from 0 to 1, a model with higher entropy has a higher precision in classifying participants in the specified number of profiles. Although entropy should not formally be used in model selection [72], it can potentially reflect the degree of accuracy in the classification.

Finally, to avoid overinterpreting the results, the best solution should take into account both profile patterns and theoretical rationale. When a solution shows profiles parallel with each other, the utility of conceptualizing the latent variable as categorical (i.e., latent profiles) rather than continuous (i.e., latent factor [46, 73–76]) is called into question. A parallel profile pattern suggests that each profile is only quantitatively different from each other. The fact that LPA arrives at a solution does not necessarily imply the existence of profiles at the population level. To our knowledge, there has been no empirical test examining whether the Dark Triad has different profiles, and if so, how many. Therefore, we rely mostly on fit indices and profile patterns to choose the best solution.

As a supplementary analysis, we modeled the data with a series of factor mixture models (FMM), which are a combination of factor analysis and LPA. While LPA assumes a latent construct (such as the Dark Triad) to be categorical in nature, it does not allow individuals within each profile to differ in degree. Similarly, factor analysis assumes a latent construct to be continuous in nature: It does not permit individuals to be qualitatively different. FMM, however, allows the same latent variable to be operationalized as both categorical (i.e., profiles that are qualitatively different from each other) and continuous (i.e., individuals who differ quantitatively in the trait of Dark Triad). We briefly report the results of FMM.

Finally, we related the CFA and best-profile solutions with external variables, including Big Five personality and SDO. Previous researchers have examined the relationship of the Dark Triad subconstructs with Big Five personality and SDO only from a variable-centered perspective [10]; thus it is unknown if the person-centered approach will show additional value. We examined how the LPA profiles differ on external variables using the approach of Bakk and Vermunt [77] (implemented in the Mplus program), and compared this approach to the variable-centered, examination-of-correlations approach.

## Results

### Latent profile analysis

**Fig 1:**
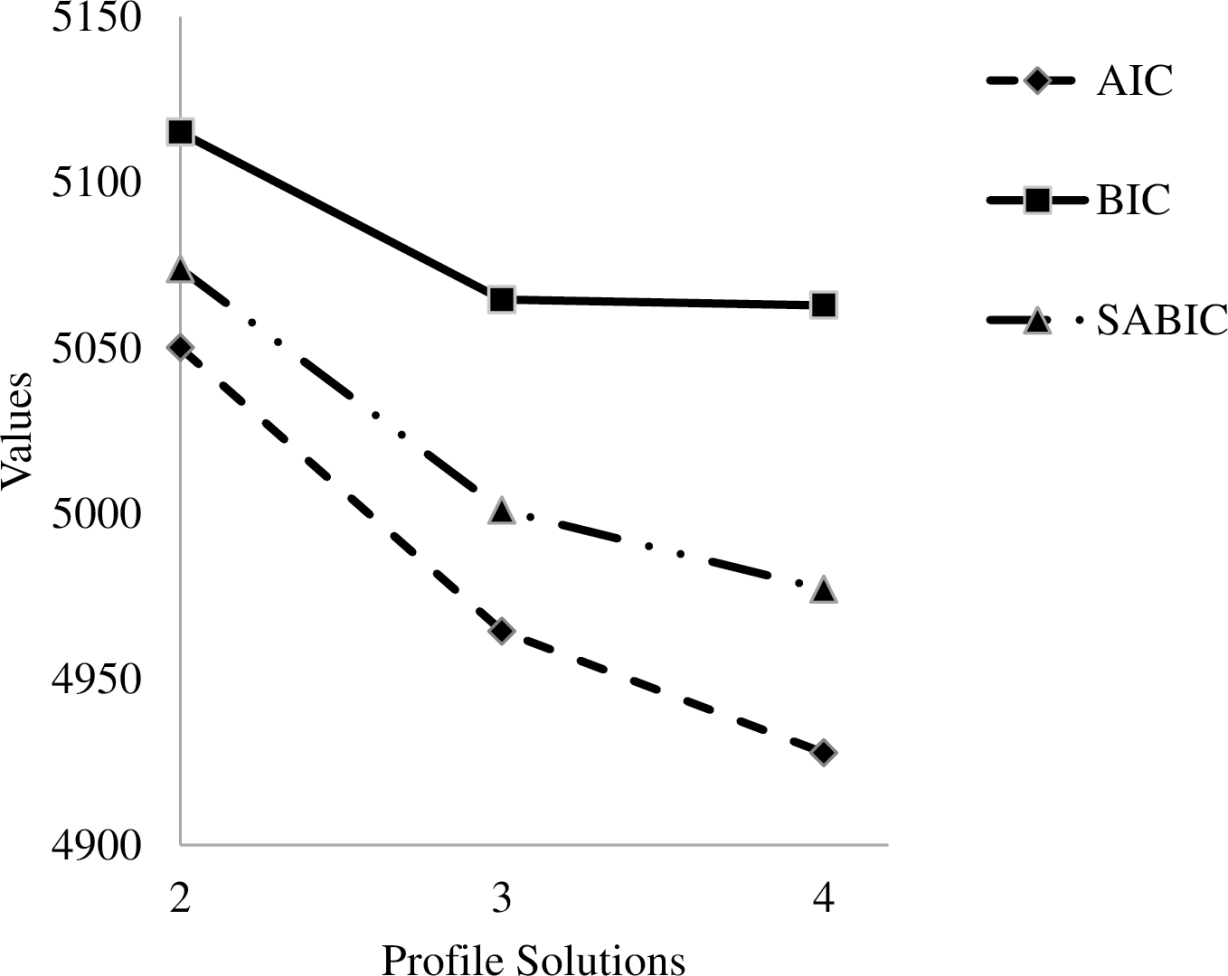
Elbow plot for loglikelihood-based indices among the LPA solutions.

We extracted a model with two profiles and then gradually increased the number of profiles to six. As shown in Table 1, the number of respondents within a given profile shrinked to about 2% as the model became more complex. To facilitate interpretation, in Fig 1 we presented “elbow plots” for AIC, BIC, and SABIC to show the gain associated with an additional profile in a solution [67]. Recall that the best solution has the lowest value on the indices. An obvious pattern was that BIC leveled off after the three-profile solution. However, the 4-profile solution identified a profile of negligible size (2.18% or 24 respondents), potentially indicating a problem of over-extracting information if more profiles were requested. Indeed, the 5- and 6-profile solutions did not converge properly even with a large number of random starts (20,000), indicating possible over-extraction. Based on BIC value, entropy and profile size, we proceeded to examine the profile pattern in the 3- and 4-profile solutions.

**Table 1:** Fit information of latent profile analysis (LPA), confirmatory factor analysis (CFA), and factor mixture modeling (FMM).

						Percentage of Respondents in Each Profile					
	AIC		BIC	SABIC	Entropy	Profile 1	Profile 2	Profile 3	Profile 4	Profile 5	Profile 6
**LPA**											
2-profile	5050.08		5115.14	5073.85	.58	53.72%	46.28%				
3-profile	4964.46		5064.56	5001.03	.65	65.43%	13.98%	20.60%			
4-profile	4927.77		5062.90	4977.14	.68	11.43%	2.18%	49.18%	37.21%		
5-profile		inadmissible solutions									
6-profile		inadmissible solutions									
**CFA**	4974.60		5019.64	4991.05	-	-	-	-	-	-	-
**FMM**											
2-profile		inadmissible solutions									
3-profile		inadmissible solutions									
4-profile		inadmissible solutions									

Note. We attempted all four types of FMM models, as described in Clark et al. [43], for each profile analysis, but all solutions were not admissible.

*N* = 1,102.

**Fig 2:**
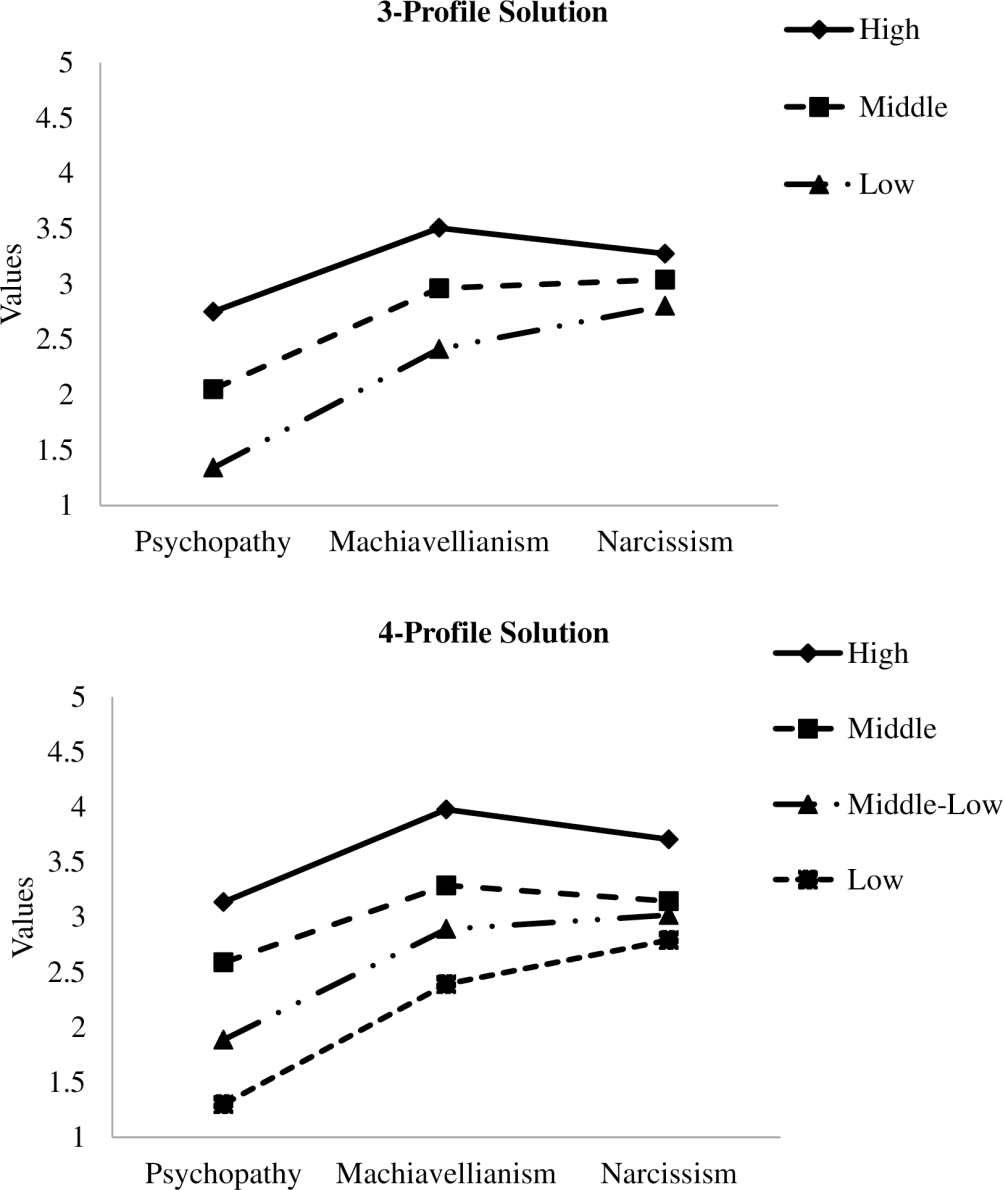
Selected latent profile solutions.

As shown in Fig 2, for both 3- and 4-profile solutions, the profiles within a solution were largely parallel: they differed only quantitatively, not qualitatively. Also apparent was that the values on narcissism were very similar for some profiles, suggesting that of the three subconstructs, narcissism was least able to differentiate the profiles. (We also inspected the shape of the 2-profile solutions; similar parallel patterns were found; results not shown here.) We concluded that the LPA solutions did not strongly support qualitatively distinct profiles in the data. To ensure this interpretation was correct, we proceeded to examine the CFA solution and compared the models in terms of their fit indices. A CFA solution with comparable or better fit would support our preliminary explication.

### Confirmatory factor analysis

CFA results replicated previous research that Machiavellianism and psychopathy had sizable standardized factor loadings on the latent variable, Dark Triad (.65 and .77), and narcissism had the weakest loading (.30). This was consistent with the previous finding that Machiavellianism and psychopathy had a closer relationship with each other than with narcissism [1]. Note that the CFA model was a just-identified model (i.e., zero degrees of freedom); thus chi-square-based fit indices, such as comparative fit index (CFI) and root mean square error of approximation (RMSEA), were not possible.

However, AIC, BIC, and SABIC were based on loglikelihood statistics and were provided in Table 1. Compared to the best LPA solutions (3-profile and 4-profile), the CFA model had the lowest BIC. Its AIC and SABIC values were lower than the 3-profile solution and but slightly higher than the 4-profile solution. Because, as noted, the LPA solutions showed largely parallel profiles (Fig 2), the data overall suggested that the Dark Triad was better represented as a dimensional than a taxonic variable.

### Factor mixture modeling

To complete the analysis, we attempted to conduct FMM, which simultaneously models the Dark Triad as a continuous variable (as in CFA) and as a categorical variable (as in LPA). According to Clark, Muthén, Kaprio, D’Onofrio, Viken, and Rose [43], the maximum number of profiles in FMM should be equal to the number of profiles in the best LPA solution. Therefore, we extracted a FMM solution with up to four profiles with all observed indicators loading on the same continuous latent factor. Unfortunately, none of the solutions converged properly, mostly because of nonpositive definite matrices. Further examination of the inadmissible FMM solutions revealed latent profiles again parallel to one another, suggesting that the analysis might be unable to separate the variance because of qualitatively distinct profiles from the variance due to the continuous latent variable. This could happen when there were actually no qualitatively meaningful profiles in the data.

We therefore concluded that the Dark Triad was best measured as a continuous variable. Based on LPA, CFA, and FMM, this was the most parsimonious interpretation of the data.

### Nomological network with Big Five personality measures and SDO

Finally, we related the Dark Triad with external variables—the Big Five and SDO—to determine whether the person-centered approach provided incremental information above the variable-centered approach (Table 2). Only results significant at α < .001 were shown because of the large sample size.

**Table 2:** Construct correlations with external variables (for variable-centered approach solution) and mean comparison among profiles on external variables (for person-centered approach solution).

	Dark Triad factor score	Psychopathy residual	Machiavellianism residual	Narcissism residual
**Variable-centered approach**				
Openness	-.10***	-.04	-.04	.17***
Conscientiousness	-.26***	-.18***	.05	.31***
Extraversion	.10	.01	-.28***	.60***
Agreeableness	-.61***	-.03	-.05	.19***
Neuroticism	.18***	.08	.06	-.31***
SDO	.53***	-.11***	.12***	-.003
	High	Middle	Low	
**3-profile solution**				
Openness	3.46	3.45	3.61	
Conscientiousness***	3.25_a_	3.46_a_	3.76_b_	
Extraversion	3.56	3.48	3.32	
Agreeableness***	3.21_a_	3.82_b_	4.13_c_	
Neuroticism***	2.92_a_	2.53_b_	2.50_b_	
SDO***	3.47_a_	2.50_b_	1.98_c_	
	High	Middle	Middle-Low	Low
**4-profile solution**				
Openness***	3.76_ab_	3.36_a_	3.49_ab_	3.68_b_
Conscientiousness***	3.56_abc_	3.19_a_	3.47_b_	3.92_c_
Extraversion	3.86	3.46	3.50	3.23
Agreeableness***	2.68_a_	3.27_b_	3.84_c_	4.24_d_
Neuroticism***	2.94_ab_	2.91_a_	2.55_b_	2.52_b_
SDO***	4.20_ab_	3.41_b_	2.50_c_	1.80_d_

*N* = 1,102.

***p < .001 for correlations (for variable-centered approach) and significantly differences among profiles (for person-centered approach). Numbers with different subscripts have statistically different means. Due to large sample size, only results with *p* < .001 are shown.

Under the variable-centered approach, we correlated the Dark Triad with those external variables. Replicating previous research, we found that the Dark Triad correlated best with agreeableness (*r* = -.61) and SDO (*r* = .53), followed by conscientiousness (*r* = -.26). Profile differences on those external variables revealed similar trends: the high Dark Triad group tended to have the lowest levels of agreeableness, and a high (though not always significantly the highest) level of SDO. The results with conscientiousness were a bit ambiguous–in both 3- and 4-profile solutions, the high Dark Triad group did not differ with other groups in terms of conscientiousness. In the 3-profile solution, the mean difference between the high and the middle groups in conscientiousness was not large (3.25 vs. 3.46). In the 4-profile solution, this result was partly because of the large estimated standard error in the high group (*SE*s = .16 in the high group vs. the average of .05 in other groups). As a result, the large standard errors were also found for the estimated means of all other external traits in the high group. Overall, the results from the variable-centered approach were consistent with those from the person-centered approach–the profile solutions showed the best ability to discriminate among groups in an external trait when the trait also had the highest correlations with the overall Dark Triad factor score.

In the four-profile solution, the exceptionally large standard error in the high group often indicated the potential problem of over-extraction. Uncertainty in estimating the means with external variables could occur when the LPA had high classification uncertainty (i.e., low entropy): The analysis could not confidently assign an individual to a group. Indeed, even for the 4-profile solution, the entropy value was quite low (.68) and rather far away from the perfect classification entropy of 1. Overall we did not see that the profile solutions provided much additional information beyond the variable-centered approach.

## Discussion and Implications

Variable-centered approaches assume the absence of qualitatively (categorically) different subpopulations in a sample. Previous researchers have often employed a variable-centered approach in their empirical examination without explicitly testing this assumption. The current research thus provides a timely addition to the literature of the Dark Triad. Despite the increasing popularity of person-centered approaches in psychological measurement (e.g., PTSD, ADHD, organizational commitment, school bullying), the present results failed to demonstrate the superiority of the person-centered approach beyond the existing variable-centered approach. Therefore, our findings failed to support recent suggestion of using person-centered approach to analyze the Dark Triad constructs [52].

There are three key findings. First, fit indices such as BIC, the best indicator to select a latent variable model, showed that LPA solutions did not fit much better than CFA solutions. Second, the LPA profile solutions showed parallel shapes, meaning that the profiles differed only quantitatively, not qualitatively. Third, the nomological network investigation showed that the profile solutions did not provide incremental useful information beyond the variable-centered solutions. Based on the principle of parsimony, the results taken together favor the variable-centered approach: Participants in the sample are quantitatively different on the Dark Triad constructs, but they are qualitatively homogeneous. This appeared to align with Jonason and Jackson’s [38] finding that all three Dark Triad traits were correlated with negative affectivity, reward sensitivity, and dysfunctional impulsivity in a similar pattern, implying that Dark Triad traits could be driven by a common set of factors. Therefore, the Dark Triad is best measured as a dimensional variable, at least with student populations that share substantial demographic characteristics such as ours (mostly Caucasian young adults who are well educated in North America).

Although our results showed that the Dark Triad traits are best conceptualized as continuous constructs, we should emphasize that our latent profile analysis has provided further insight to the nature of the Dark Triad. Our latent profile results (Fig 2) revealed clearly that individuals high in one member trait of the Dark Triad (e.g., psychopathy) also tended to score high in other traits. This finding is consistent with our CFA results, in which each trait (particularly Machiavellianism and psychopathy) has a sizeable factor loading on the overall latent construct of Dark Triad. Even though researchers may occasionally make person-centered predictions (e.g., individuals high on the Dark Triad will do A), the Dark Triad traits are closely related to each other rather than being three distinct variables. Therefore, it may be appropriate to make inferences to individuals by modeling the overall latent construct of Dark Triad using a CFA procedure, rather than treating the three Dark Triad traits as distinct variables. The overall latent construct would represent individuals’ standing on this construct.

### Limitations

As with any research, the current research has certain limitations. First, as mentioned, our sample is predominantly Caucasian university students studying in North America. Due caution should be taken before generalizing the results to other populations. On the other hand, because the Dark Triad concept emerged mainly from research with subclinical populations, the current sample provides continuity with previous research. Still, for researchers who are interested in studying the Dark Triad constructs at the clinical level, our results may not generalize to this specific population. Further LPA and FMM research using both clinical and nonclinical populations is required to determine the relative merits of person- vs. variable-centered (or hybrid) approaches.

A second limitation is the current study’s reliance on self-report data. When people try to tell others about themselves, a certain amount of self-presentation bias is inevitable: He and van de Vigner [78] confirmed the existence of self-presentation bias in self-report data across domains, including personality. Because Dark Triad items are probably vulnerable to such bias [39], we must move beyond self-rating measures to concrete behavior [79] when examining the Dark Triad.

Finally, a few researchers have begun to study the Dark Tetrad—the Dark Triad plus sadism. Sadism has been shown to predict social aversive behaviors above and beyond those Dark Triad subconstructs [2, 80–82]. Future research may continue to examine the utility of incorporating sadism into the Dark Triad and to examine the data structure of sadism using both variable- and person-centered approaches.

## Conclusion

Allport [49], back in 1937, insightfully pointed out psychological processes as a coordinated rather than isolated system, and thus it may be appropriate to use individuals with various profile configurations, not independent traits, as the unit of analysis. The current research followed Allport’s recommendation to examine the appropriate analytic approach for the Dark Triad. On one hand, LPA solutions showed quantitatively parallel profiles, meaning that individuals scoring high on one trait are very likely to possess other traits' qualities. The Dark Triad traits should thus be studied using variable-centered approach. On the other hand, LPA solutions suggested that the three traits may not be three distinct characteristics because individuals scoring high on one trait also tend to be strong in other Dark Triad traits. Thus our results supported the modeling of an overall, higher-level construct (with confirmatory factor analysis, for example) to represent individuals’ standing on the Dark Triad (i.e., making person-centered predictions). Finally, we reiterate that the assumption was examined only in a North American student population. Thus this paper may act as an example of how the assumption may be tested in another population.
